# Effects of repeated binge intake of the pyrovalerone cathinone derivative 3,4-methylenedioxypyrovalerone on prefrontal cytokine levels in rats – a preliminary study

**DOI:** 10.3389/fnbeh.2023.1275968

**Published:** 2023-11-10

**Authors:** Erin K. Nagy, Jonna M. Leyrer-Jackson, Lauren E. Hood, Amanda M. Acuña, M. Foster Olive

**Affiliations:** ^1^Department of Psychology, Behavioral Neuroscience and Comparative Psychology Area, Arizona State University, Tempe, AZ, United States; ^2^Department of Medical Education, School of Medicine, Creighton University, Phoenix, AZ, United States; ^3^Interdisciplinary Graduate Program in Neuroscience, School of Life Sciences, Arizona State University, Tempe, AZ, United States

**Keywords:** pyrovalerone cathinone derivative, methylenedioxypyrovalerone, psychostimulant, self-administration, binge access, rat, prefrontal cortex

## Abstract

Drugs of abuse activate neuroimmune signaling in addiction-related regions of the brain, including the prefrontal cortex (PFC) which mediates executive control, attention, and behavioral inhibition. Traditional psychostimulants including methamphetamine and cocaine are known to induce PFC inflammation, yet the effects of synthetic cathinone derivatives are largely unexplored. In this study, we examined the ability of repeated binge-like intake of the pyrovalerone cathinone derivative 3,4-methylenedioxypyrovalerone (MDPV) to alter cytokine profiles in the PFC. Male and female rats were allowed to intravenously self-administer MDPV (0.05 mg/kg/infusion) or saline as a control under conditions of prolonged binge-like access, consisting of three 96 h periods of drug access interspersed with 72 h of forced abstinence. Three weeks following cessation of drug availability, PFC cytokine levels were assessed using antibody arrays. Employing the unsupervised clustering and regression analysis tool CytoMod, a single module of co-signaling cytokines associated with MDPV intake regardless of sex was identified. With regards to specific cytokines, MDPV intake was positively associated with PFC levels of VCAM-1/CD106 and negatively associated with levels of Flt-3 ligand. These findings indicate that prolonged MDPV intake causes changes in PFC cytokine levels that persist into abstinence; however, the functional ramifications of these changes remain to be fully elucidated.

## Introduction

Most drugs of abuse, including psychostimulants, activate neuroimmune signaling mechanisms in the brain (Lucerne et al., 2021; Namba et al., 2021). This occurs via several mechanisms, including activation of peripheral immune cells which in turn alter brain function via the neuroimmune axis. Central nervous system inflammation can also be caused by drug action within the brain itself, such as activation of toll-like receptors, complex interactive neuron-glia processes, and the formation of cell damaging reactive oxygen or nitrogen species. Activation of neuroinflammatory signaling can alter normal brain function, and may contribute to some of the hallmark features of addiction such as deficits in cognitive flexibility, loss of inhibitory control, maladaptive memory formation, and insensitivity to negative consequences (Crews et al., 2011; Correia et al., 2020; Escobar et al., 2023), many of which are governed by the prefrontal cortex (PFC). Indeed, a number of studies have shown that repeated exposure to or intake of psychostimulants lead to increased PFC levels of pro-inflammatory cytokines such as interleukins 1β and 6 (IL-1β and IL-6, respectively) and tumor necrosis factor alpha (TNFα) (Goncalves et al., 2008; Seney et al., 2021; Wang et al., 2021; Varodayan et al., 2023).

While most prior research on psychostimulant-induced neuroinflammation has focused on effects of cocaine and amphetamine-type stimulants, relatively little is known about the effects of newly developed synthetic cathinone derivatives, frequently referred to as “bath salts.” Such cathinone analogues include methylmethcathinone (mephedrone), α-pyrrolidinopentiophenone (α-PVP), and the pyrovalerone-based derivative 3,4-methylenedioxypyrovalerone (MDPV), which have potent cocaine or methamphetamine-like mechanisms of action. Prior studies examining effects of these cathinone derivatives on central inflammatory markers are scant, and of those that have been published, results thus far have been mixed. For example, repeated passive administration of MDPV, a potent and long-lasting monoamine reuptake inhibitor, to rats for 9 days followed by 72 h of withdrawal did not alter PFC levels of IL-1β, IL-6, IL-10, IL-17A, chemokine C-C ligand 2 (CCL2), CCL5, or TNF-α (Inan et al., 2023). However, a separate study showed that voluntary self-administration of α-PVP, which has neurochemical mechanisms of action similar to those of MDPV, resulted in increases in PFC levels of IL-1α, IL-1β, IL-6, and TNF-α when assessed the day following cessation of drug intake (Marusich et al., 2022). These effects were particularly evident in male animals with longer periods of daily drug access (i.e., 6 h/day). These disparate results are not surprising, given the highly dynamic nature of neuroinflammatory signaling and likely contributing roles of active drug self-administration across numerous hours per day vs. passive administration of a single dose by an experimenter. Some of these changes may be functionally relevant for addiction-related behaviors, as it has been demonstrated that pharmacological blockade of CXCR4 attenuates the conditioned rewarding effects of MDPV (Oliver et al., 2018), and blockade of CXCR4, CCR5, or IL-17A signaling attenuates MDPV-induced alterations in anxiety-like behaviors (Simmons et al., 2022; Inan et al., 2023).

The purpose of the present study was to assess the effects of repeated binge-like intake of the pyrovalerone cathinone MDPV in rats on PFC levels of cytokines as an index of neuroinflammation. Of particular note, some synthetic cathinone users abuse the drug in binge-like patterns over the course of several days, followed by periods of abstinence (Miotto et al., 2013; Palamar et al., 2015). To mimic this pattern of intake, we utilized a self-administration paradigm consisting of extended (96 h) drug access periods that are followed by 72 h of forced abstinence in the home cage. Following this initial phase, the process is repeated several times to model multiple binge-abstinent episodes (Cornett and Goeders, 2013). We previously used this paradigm to demonstrate neurocognitive impairment and neurodegeneration in the entorhinal cortex following MDPV intake (Sewalia et al., 2018). The present study represents a preliminary investigation into changes in cytokine levels in the PFC that occur following MDPV intake and a period of abstinence, which may alter a number of PFC-mediated cognitive and behavioral functions, though none were specifically assessed in this study. To determine any effects that persist beyond initial abstinence, which is the time frame most frequently assessed in other studies of psychostimulant-induced neuroinflammation (Lucerne et al., 2021; Namba et al., 2021), we allowed a 3 weeks period of abstinence in the home cage prior to brain harvest. Assessment of relative levels of 79 different cytokines was performed using capture antibody arrays.

## Methods

### Animals

A total of 27 adult (*n* = 13 male, *n* = 14 female) Sprague–Dawley rats (300–350 g, Envigo, Placentia, CA) were used as subjects. Prior to implantation of venous catheters, animals were pair housed in a vivarium on a reversed light–dark cycle (12:12; lights off at 0700 h), with temperature and humidity within guidelines of the National Institutes of Health *Guide for the Care and Use of Laboratory Animals* (8th edition). Following surgical procedures, rats were single housed to prevent cagemate chewing and damage to vascular access ports. Food and water were available *ad libitum* at all times. All animal practices and procedures outlined here were approved by the Arizona State Institution Animal Care and Use Committee (IACUC).

### Drugs

MDPV was provided by the National Institute on Drug Abuse Drug Supply Program (Research Triangle Park, NC), and was dissolved in 0.9% w/v sodium chloride for intravenous self-administration.

### Surgical procedures

Rats were anesthetized with isoflurane (5% induction, 2–3% maintenance) vaporized in oxygen at a flow rate of 2 L/min. The jugular vein was then exposed on the ventral side of the neck, and silastic catheters were inserted ~3.0 cm into the vein and secured in place with sutures. The other end of the catheter was routed subcutaneously to exit the skin between the scapulae. The catheter was connected to a vascular access port (Instech Laboratories, Plymouth Meeting, PA) and secured to the surrounding skin with sutures. Following implantation, access ports were flushed with 0.2 mL of an antibiotic solution containing 66.6 mg/mL ticarcillin/clavulanic acid dissolved in sterile saline containing 70 U/mL heparin. During postoperative care (5 days), rats received daily infusions of this solution to maintain catheter patency, and during the first 3 days rats were administered meloxicam (2 mg/kg s.c.) and buprenorphine (0.03 mg/kg s.c.) daily to reduce post-surgical discomfort. Animals were then randomly assigned to commence training for intravenous self-administration of either MDPV (*n* = 8 per sex) or saline (*n* = 5 males and *n* = 6 females) as controls. Catheter patency was verified once weekly by infusion of the short-acting barbiturate Brevital (10 mg/kg) and observation of brief periods of immobility.

### Apparatus

Self-administration procedures were performed in operant conditioning chambers (Med Associates, St. Albans, VT) interfaced to a PC computer. Located in each chamber were a 2.5 cm diameter active nosepoke aperture and a similarly sized inactive aperture. A stimulus light was located above both apertures to provide a visual cue during each drug infusion, and atop each chamber was a speaker that provided an auditory tone (~65 dB, 2,900 Hz) during each drug delivery. Food pellets were placed on the floor of the chamber every morning of each self-administration, and a water bottle was always available. Intravenous solutions (MDPV or saline) were delivered to a liquid swivel mounted above the chamber via a PC-controlled syringe pump and polyethylene tubing. Attached to the swivel was a protective metal spring tether that contained polyethylene tubing connected to the vascular access port. Each chamber was placed in a separate sound-attenuating cubicle equipped with a house light programmed to match the light–dark cycle of the colony room, and a ventilation fan to mask external noise and odors.

### Self-administration procedures

Rats were allowed to spontaneously acquire self-administration of MDPV or saline in 96 h sessions. A dose of 0.05 mg/kg/infusion for MDPV was selected based on our prior studies where we have observed reliable self-administration in rats under both daily limited access and 96 h binge-like access conditions (Watterson et al., 2014; Sewalia et al., 2018; Nagy et al., 2020). After the first 96 h session, animals were returned to the home cage for 72 h of abstinence, and this procedure was repeated twice, so that each animal underwent a total of three 96 h self-administration sessions, each separated by 72 h of abstinence in the home cage. All reinforcers were available on a fixed-ratio 1 (FR1) schedule of reinforcement. Nosepokes into the designated active aperture resulted in reinforcer delivery in a volume of 0.06 mL over a 1 s period, which was accompanied by concurrent illumination of the stimulus light and presentation of the auditory tone. Following each infusion, a 20 s timeout period was enacted where additional active nosepokes were recorded but had no consequences. Nosepokes into the designated inactive aperture had no consequences at any time. Prior to and following each 96 h session, catheters were flushed with 0.1 mL of antibiotic solution. Acquisition criteria for MDPV self-administration required at least 50 infusions to be acquired during the first 96 h session. Yoked administration of saline was not utilized as a control since such non-contingent delivery of drug or saline solutions can have aversive properties and reduce motivation for intake of psychostimulants (Twining et al., 2009).

Following the last (third) 96 h session, rats remained undisturbed in the home cage for 3 weeks. This period of abstinence was selected to allow for complete elimination of MDPV and its metabolites, examination of lasting changes in markers of neuroinflammation that persist beyond the acute withdrawal phase, and to be in alignment with our previous findings of MDPV-induced cognitive deficits and neurodegeneration at this time point in the 96 h binge paradigm (Sewalia et al., 2018). During this time, catheters were not flushed and the only contact with an experimenter occurred during once weekly cage changes. Of the *n* = 8 animals per sex assigned to self-administer MDPV, *n* = 2 of each were removed from the study following the self-administration phase due to development of health complications possibly due to adverse cardiovascular complications induced by MDPV (Coppola and Mondola, 2012; McClenahan et al., 2019). No animals in the saline groups required removal from the study.

### Brain cytokine arrays

Animals were euthanized with CO_2_ asphyxiation followed by rapid decapitation and removal of the brain, rinsing with chilled 1× phosphate buffered saline (PBS) and placement onto a chilled dissection platform. The PFC of each animal was dissected and placed separately into a microcentrifuge tube containing 0.5 mL ice-cold 1× PBS containing 50 μL each of 100× protease and phosphatase inhibitors (ThermoFisher, Waltham, MA). Tissues were then homogenized using a Branson sonicator and stored immediately at −80°C. Total protein content of each sample was determined by bicinchoninic acid (BCA) assay (ThermoFisher) following the manufacturer’s directions. For determination of cytokine levels, a Proteome Profiler Rat XL Cytokine Array (ARY030, R&D Systems, Minneapolis, MN) was utilized. This assay measures relative levels of 79 different cytokines in duplicate with membrane immobilized antibodies. Each membrane was loaded with 200 μg of total protein. Results were visualized with WesternSure Enhanced Chemiluminescence (ECL) Substrate on a C-Digit ECL scanner (Li-Cor, Lincoln, NE). Pixel intensity values of each pair of dots representing different antibodies to cytokines were analyzed using QuickSpots software (Ideal Eyes Systems, Bountiful, UT) as recommended by the manufacturer. For each array, the average pixel intensities of two negative control spots were considered to be background signal, and subtracted from each of the average pixel values for target proteins.

### Statistical analyses – self-administration data

A three-way (drug condition × sex × session) mixed model analysis of variance (ANOVA) was used to analyze the number of MDPV or saline infusions obtained by each sex during each 96 h session, followed by Bonferroni’s correction for multiple comparisons. As no significant main effect of sex was observed (see Results), total intake values were collapsed across sex and analyzed by two-way ANOVA, with session and drug condition as factors, followed by Bonferroni’s multiple comparisons. value of *p*-values less than 0.05 were considered statistically significant. GraphPad Prism v.10.0 (GraphPad Software, La Jolla, CA) was utilized for these analyses.

### Statistical analyses – cytokine levels

For analysis of cytokine levels, we initially performed a three-way ANOVA with sex, drug condition, and cytokine as factors. However, these analyses revealed that cytokine measurement data were not normally distributed and failed D’Agostino and Pearson, Anderson-Darling, Shapiro–Wilk, and Kolmogorov–Smirnov tests for normality and lognormality. Therefore, we analyzed the cytokine dataset with CytoMod, an open source Python based analysis tool that utilizes unsupervised hierarchical clustering and regression analyses to identify clusters (“modules”) of cytokines that co-vary in expression levels (Cohen et al., 2019). This method offers numerous advantages over parametric approaches, including increased statistical power due to reduced need for multiplicity adjustments following thousands of statistical comparisons, assessment of cytokines levels relative to other similarly varying cytokines, and potential identification of co-signaling cytokines without any *a priori* assumptions or hypotheses regarding cytokine function.

Drug condition (MDPV or saline) and sex (male or female) were input as covariates. Pixel intensity values for each cytokine, which are linearly related to cytokine concentration, were first regressed against the mean value for that cytokine, and adjusted values were defined as the residuals from the regression. Complete linkage agglomerative hierarchical clustering was then used to form modules of individual cytokines using Pearson’s correlation coefficient as a distance metric. Due to inherent sensitivity of this approach to minor perturbations of the dataset, a repeated bootstrap clustering method (Dudoit and Fridlyand, 2003) utilizing 1,000 randomly perturbed data sets was performed to increase cluster robustness. Final hierarchical clustering was performed on the resulting matrix of reliability fractions, and the number of clusters (K) for each dataset was determined with the Tibshirani gap statistic (Tibshirani et al., 2001). The optimal *K* number was selected based on when the gap statistic first achieved a positive value. To visualize cytokine classification in each module, a Kernel principal components analysis (PCA) was performed. Pairwise Pearson’s correlations between cytokines following adjustment to the mean values were generated and sorted along both axes using complete linkage and dendrogram annotation. A heatmap of cytokine modules over Pearson pairwise correlation similarity measures was then generated and clustered cytokines into *K* modules. Final modules were then constructed by clustering pairwise reliability scores. Two output tables were generated, one containing the odds ratio (OR), adjusted value of *p*s, family-wise error rates (FWER, adjusted using the Bonferroni–Holm method) and false discovery rates (FDR, adjusted using the Benjamini Hochberg procedure). These tables show significant associations of a specific cytokine module with MDPV intake (Table 1), and each individual cytokine that was significantly associated with a particular covariate (Table 2). Associations with cytokine modules or individual cytokines were considered significant only if value of *p*s were less than 0.05.

**Table 1:** List of identified cytokine modules and their association with MDPV intake.

Covariate	Module	Odds ratio	*p*-value	FWER	FDR
MDPV	**M5**	**9.7**	**0.0355**	**0.178**	**0.178**
MDPV	M3	0.689	0.407	1	0.822
MDPV	M4	0.802	0.593	1	0.822
MDPV	M1	0.835	0.658	1	0.822
MDPV	M2	0.941	0.882	1	0.882

Bolded text indicates statistically significant association with the specified covariate.

**Table 2:** List of all cytokines analyzed in the present study and their statistical associations with MDPV intake.

Covariate	Cytokine	Odds ratio	*p*-value	FWER	FDR
**MDPV**	**VCAM-1/CD106**	**8.4**	**0.0151**	**1**	**0.877**
**MDPV**	**Flt-3 Ligand**	**0.254**	**0.0333**	**1**	**0.877**
MDPV	RGM-A	10.4	0.0578	1	0.877
MDPV	Neprilysin/CD10	14.1	0.0611	1	0.877
MDPV	IL-3	0.361	0.0707	1	0.877
MDPV	Fibulin 3	0.43	0.1	1	0.877
MDPV	LIF	0.455	0.11	1	0.877
MDPV	Cyr61/CCN1	0.368	0.113	1	0.877
MDPV	IGF-I	2.23	0.127	1	0.877
MDPV	Prolactin	0.469	0.141	1	0.877
MDPV	FGF-7/KGF	1.92	0.154	1	0.877
MDPV	TWEAK/TNFSF12	0.486	0.164	1	0.877
MDPV	MMP-3	0.546	0.188	1	0.877
MDPV	GDF-15	0.534	0.203	1	0.877
MDPV	CCL17/TARC	0.538	0.204	1	0.877
MDPV	CCL21/6Ckine	1.73	0.229	1	0.877
MDPV	CCL2/JE/MCP-1	1.72	0.23	1	0.877
MDPV	IL-17A	1.87	0.246	1	0.877
MDPV	IL-2	0.591	0.246	1	0.877
MDPV	Galectin-3	1.76	0.247	1	0.877
MDPV	Galectin-1	0.602	0.25	1	0.877
MDPV	IL-13	0.613	0.268	1	0.877
MDPV	Jagged 1	1.71	0.298	1	0.877
MDPV	Adiponectin/Acrp30	0.631	0.321	1	0.877
MDPV	Pref-1/DLK1/FA1	1.59	0.322	1	0.877
MDPV	IL-22	1.57	0.332	1	0.877
MDPV	NOV/CCN3	0.65	0.334	1	0.877
MDPV	IL-1ra/IL-1F3	0.658	0.361	1	0.877
MDPV	RBP4	1.48	0.364	1	0.877
MDPV	VEGF	1.52	0.371	1	0.877
MDPV	Osteoprotegerin/TNFRSF11B	0.684	0.388	1	0.877
MDPV	NT-4	0.7	0.394	1	0.877
MDPV	Cystatin C	0.703	0.395	1	0.877
MDPV	WISP-1/CCN4	1.43	0.398	1	0.877
MDPV	Resistin	1.43	0.399	1	0.877
MDPV	SCF	1.45	0.43	1	0.877
MDPV	Hepassocin	0.712	0.44	1	0.877
MDPV	IL-1α/IL-1F1	0.727	0.444	1	0.877
MDPV	MAG/siglec-4α	1.37	0.448	1	0.877
MDPV	TNF-α	0.738	0.468	1	0.877
MDPV	IL-1β/IL-1F2	0.743	0.476	1	0.877
MDPV	MMP-9	1.36	0.477	1	0.877
MDPV	Fetuin A/AHSG	0.75	0.487	1	0.877
MDPV	IFN-γ	1.34	0.488	1	0.877
MDPV	CCL20/MIP-3α	1.31	0.522	1	0.880
MDPV	TIM-1/KIM-1/HAVCR	0.77	0.538	1	0.880
MDPV	HGF	0.763	0.54	1	0.880
MDPV	DPPIV/CD26	0.768	0.547	1	0.880
MDPV	CX3CL1/Fractalkine	1.28	0.555	1	0.880
MDPV	IL-4	0.781	0.557	1	0.880
MDPV	IL-6	0.797	0.583	1	0.887
MDPV	CNTF	1.24	0.6	1	0.887
MDPV	Serpin E1/PAI-1	0.811	0.615	1	0.887
MDPV	RAGE	0.81	0.615	1	0.887
MDPV	CCL5/RANTES	1.22	0.637	1	0.887
MDPV	EGF	0.815	0.638	1	0.887
MDPV	FGF acidic	1.2	0.659	1	0.887
MDPV	CCL3/CCL4/MIP-1α/β	1.2	0.667	1	0.887
MDPV	Endostatin	0.836	0.676	1	0.887
MDPV	PDGF-BB	1.18	0.684	1	0.887
MDPV	Clusterin	0.847	0.69	1	0.887
MDPV	CCL11/Eotaxin	0.859	0.709	1	0.887
MDPV	MMP-2	0.861	0.713	1	0.887
MDPV	IGFBP-6	0.862	0.719	1	0.887
MDPV	LIX	0.869	0.73	1	0.887
MDPV	NT-3	1.13	0.768	1	0.919
MDPV	IGFBP-3	1.11	0.803	1	0.947
MDPV	G-CSF	0.908	0.817	1	0.950
MDPV	FGF-21	0.922	0.841	1	0.962
MDPV	GM-CSF	1.08	0.853	1	0.962
MDPV	IGFBP-5	1.07	0.87	1	0.967
MDPV	Osteopontin	1.06	0.892	1	0.967
MDPV	CXCL7/Thymus Chemokine-1	1.06	0.893	1	0.967
MDPV	CXCL2/GROβ/MIP-2/CINC-3	1.05	0.911	1	0.973
MDPV	EG-VEGF/PK1	0.978	0.956	1	0.989
MDPV	Lipocalin-2/NGAL	0.979	0.96	1	0.989
MDPV	IGFBP-2	0.982	0.964	1	0.989
MDPV	CCL22/MDC	1.01	0.983	1	0.995
MDPV	ICAM-1/CD54	∞	0.999	1	0.999

Bolded text indicates statistically significant association with the covariate.

## Results

### MDPV self-administration

**Figure 1:**
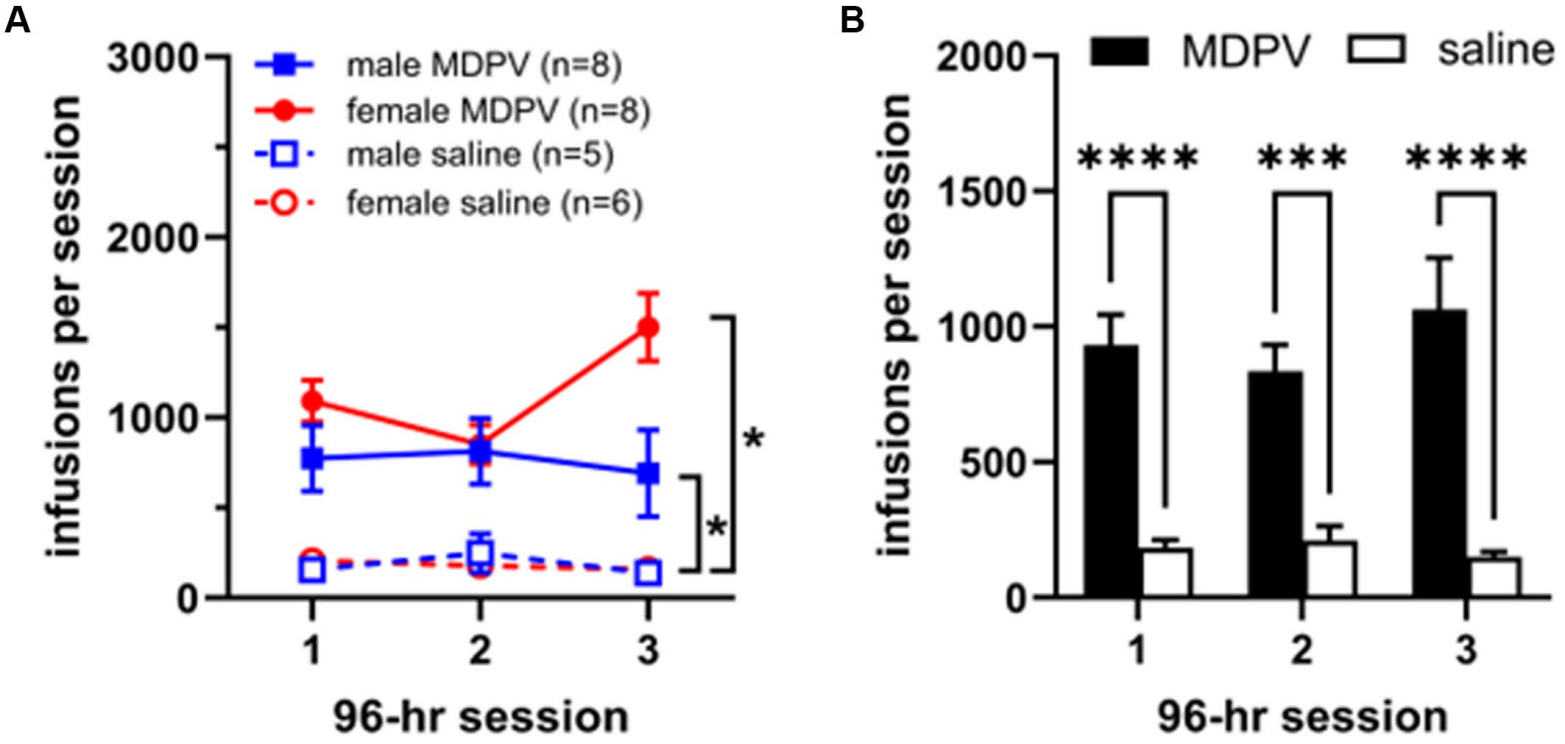
**(A)** Intake levels of MDPV and saline in male and female rats during each of three 96 h self-administration sessions. *indicates *p* < 0.05 vs. respective saline controls. **(B)** Total number of infusions of MDPV or saline obtained across each of the three 96 h self-administration sessions, collapsed across sex. *** and **** indicate *p* < 0.001 and *p* < 0.0001 vs. saline, respectively.

Three-way ANOVA analyses revealed a significant main effect of drug condition (MDPV or saline, *F*_1,23_ = 42.01; *p* < 0.0001), with animals receiving significantly more MDPV than saline infusions during all 3 sessions (Figure 1A). No main effects of sex (*F*_1,23_ = 2.13, *p* > 0.05) or session (*F*_2,40_ = 0.89; *p* > 0.05) were observed. In addition, no significant interactions of drug condition × session, (*F*_2,41_ = 2.46, *p* > 0.05), sex × drug condition (*F*_1,23_ = 2.10, *p* > 0.05), sex × session (*F*_2,41_ = 3.19, *p* > 0.05), or drug condition × sex × session (*F*_2,41_ = 1.91, *p* > 0.05) were observed. Post-hoc comparisons revealed that MDPV intake was higher than that of saline regardless of sex or session (Figure 1A). When self-administration data were collapsed across sex (Figure 1B), an effect of drug condition was observed (*F*_1,70_ = 70.56, *p* < 0.0001) but no effect of session (*F*_2,70_ = 0.28, *p* > 0.05) nor a session × drug treatment interaction (*F*_2,70_ = 0.83, *p* > 0.05).

### Changes in cytokine levels

**Figure 2:**
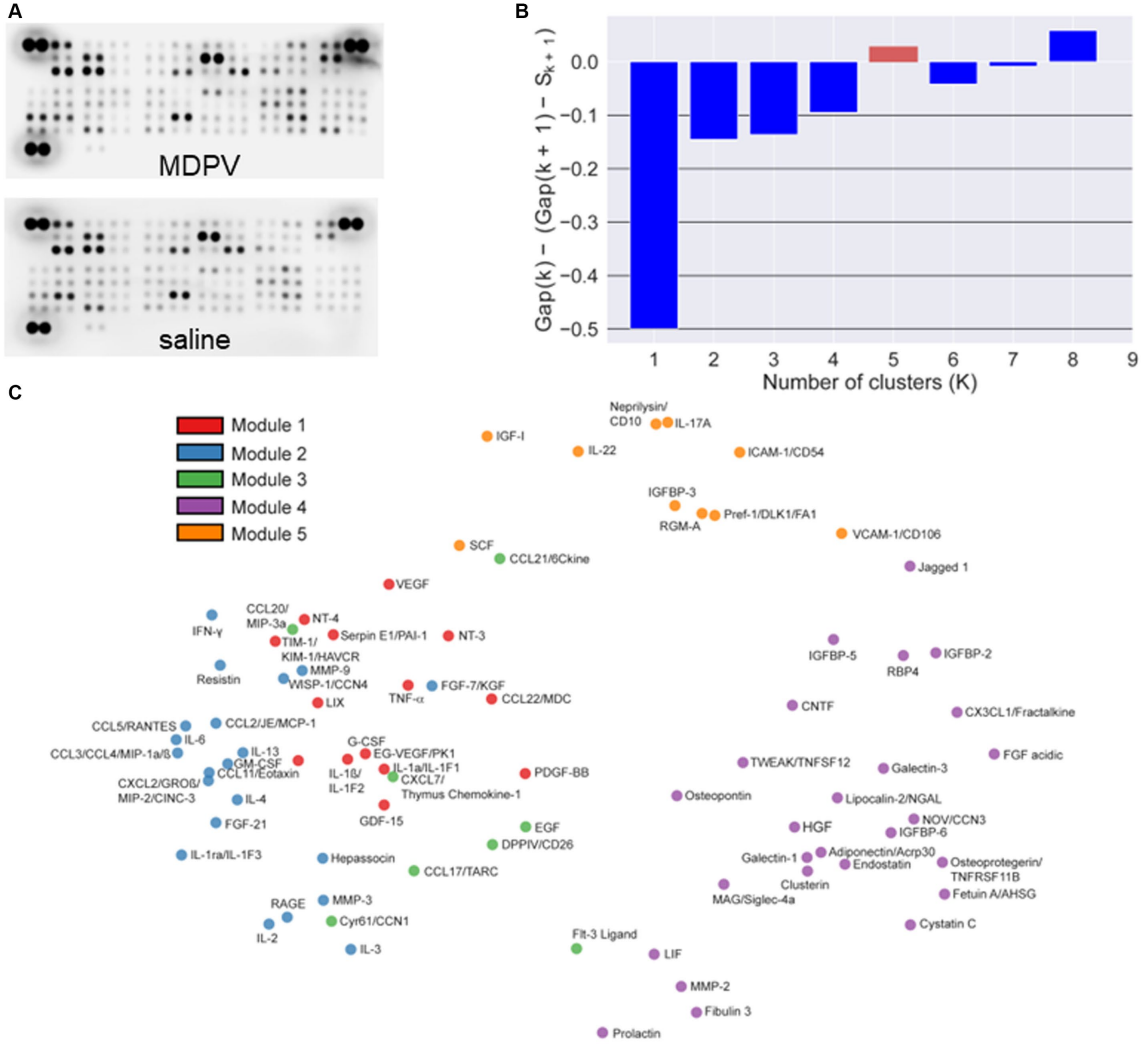

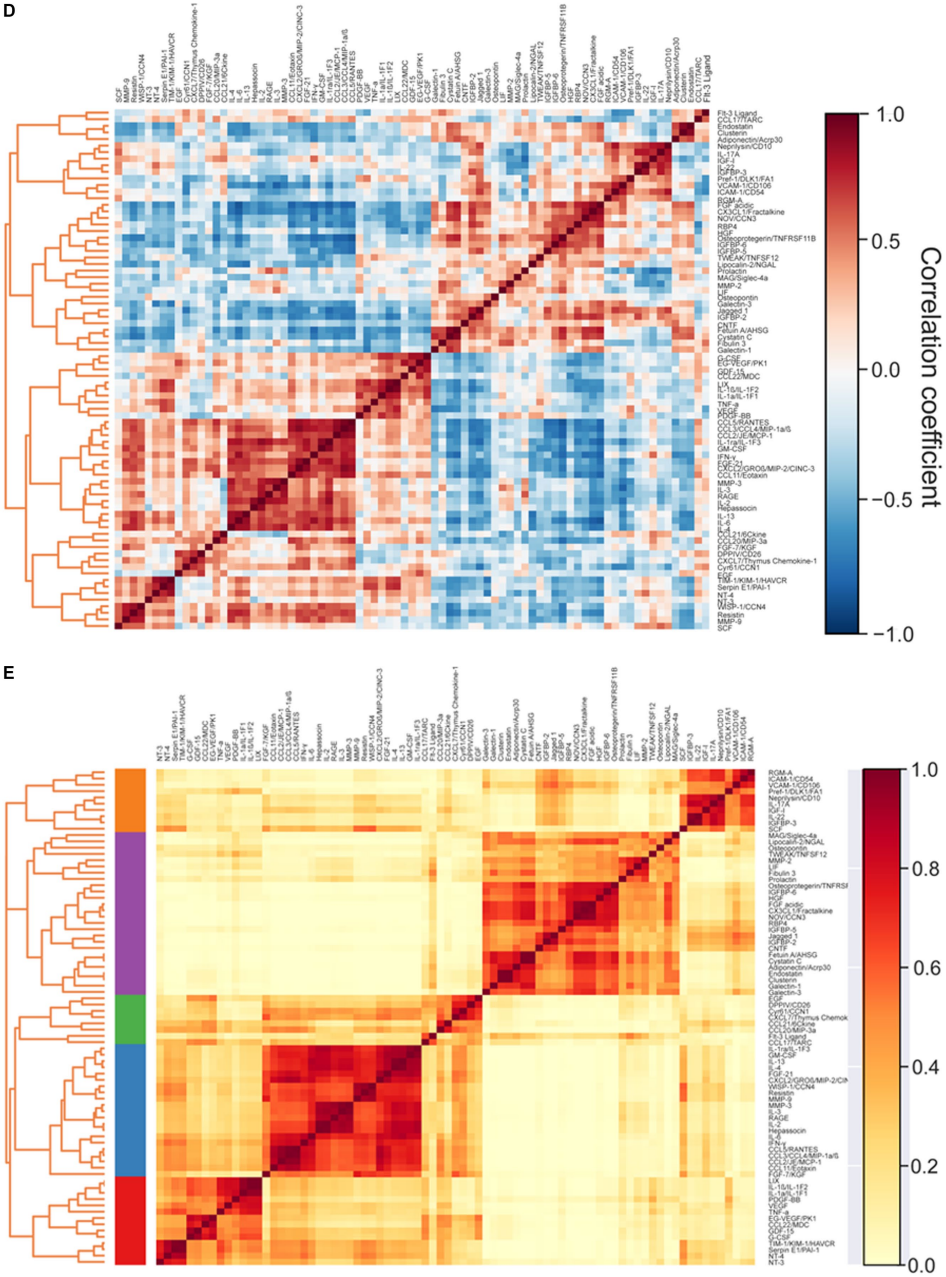
**(A)** Representative cytokine arrays incubated with PFC homogenates from animals self-administering MDPV or saline. The dense pairs of spots in the top left and right corners and the lower left corner are reference spots for alignment of the array in the analysis software. **(B)** Automated selection of the optimal number of cytokine clusters (modules, *K* = 5) via the point at which gap statistic value becomes positive (red column). **(C)** PCA analyses showing the five identified modules and their cytokine components, color coded for ease of visualization. **(D)** Pairwise Pearson’s correlation between cytokine levels. Color coding of correlation coefficients is shown on the right. Cytokines are sorted along both axes using complete linkage. **(E)** Heat map of complete linkage clustered modules over Pearson’s pairwise correlation similarity measure (color coded on right side). Modules are identified by color codes on the left side and placed in ascending order (Modules 1–5) from bottom to top along the *y*-axis.

Representative arrays for animals self-administering saline or MDPV are shown in Figure 2A. Based on resulting gap statistic calculations, a total of *K* = 5 modules were identified (Figure 2B). Classification of individual cytokines to each specific module is shown in the PCA plot in Figure 2C. The resulting pairwise Pearson correlation map between cytokines is shown in Figure 2D. The resulting heat map of the Pearson correlation similarity measure is shown in Figure 2E, where 5 distinct color-coded clusters can be observed.

These analyses revealed that a single cluster (Module 5) was associated with MDPV vs. saline self-administration (Table 1, OR = 9.7, *p* = 0.0355, FWER = 0.178, FDR = 0.178). Cytokines in Module 5 were identified as intercellular adhesion molecule 1/cluster of differentiation 54 (ICAM-1/CD54), insulin-like growth factor I (IGF-I), insulin-like growth factor binding protein 3 (IGFBP-3), IL-17A, IL-22, neprilysin/CD10, preadipocyte factor 1/delta-like 1 (Pref-1/DLK1), repulsive guidance molecule A (RGM-A), stem cell factor (SCF), and vascular cell adhesion molecule 1/CD106 (VCAM-1/CD106). Of these, only VCAM-1/CD106 was positively associated with MDPV intake (OR = 8.4, *p* = 0.0151, FWER = 1, FDR = 0.877) (Table 2). Levels of two other cytokines in Module 5, RGM-A (OR = 10.4, *p* = 0.0578, FWER = 1, FDR = 0.877) and neprilysin/CD10 (OR = 14.1, *p* = 0.0611, FWER = 1, FDR = 0.877) showed trends towards a positive statistical association with MDPV intake, but were not further analyzed. In contrast, there was a significant negative association between levels of the Module 2 cytokine Fms-related tyrosine kinase 3 (Flt-3) ligand and MDPV intake (OR = 0.254, *p* = 0.0333, FWER = 1, FDR = 0.877), and a trend towards a negative statistical association between MDPV intake and levels of IL-3 (OR = 0.361, *p* = 0.0707, FWER = 1, FDR = 0.877). However, due to failure to meet significance criterion, IL-3 was not analyzed further.

Inclusion of sex as a co-variate in the analyses did not produce any significant associations with any specific cytokine module or individual cytokine (all value of *p*s >0.05) in either saline or MDPV self-administering animals. These findings are consistent with the lack of sex differences observed in MDPV intake.

## Discussion

We observed robust intravenous self-administration of MDPV in each of the three 96 h binge-like intake periods in both males and females, similar to our previous findings using this paradigm (Sewalia et al., 2018; Nagy et al., 2020), as well findings from various laboratories under more limited access conditions (i.e., 1–6 h/day) (Aarde et al., 2013; Watterson et al., 2014; Aarde et al., 2015; Gannon et al., 2017; Doyle et al., 2021; Abbott et al., 2022). As expected, saline self-administration was minimal across all sessions. These findings confirm that the 96 h self-administration paradigm, originally designed to mimic prolonged binge-like intake of methamphetamine (Cornett and Goeders, 2013), results in robust intake of MDPV. Overall sex differences in total MDPV intake were not observed, in agreement with a previous study (Abbott et al., 2022). Prior studies examining psychostimulant-induced neuroinflammation have tended to examine such processes in close temporal proximity with active drug intake (i.e., within 1 week following cessation drug intake). Therefore, we chose to examine brain tissue following a 3 weeks post-drug or saline period of abstinence in order to determine more persistent changes. We specifically attempted to minimize potential external confounds by allowing animals to remain undisturbed this time, with no experimenter interaction, albeit with the exception of weekly cage changes. While we cannot rule out the contribution of potential unknown environmental events, or effects of loss of catheter patency during this time, such influences would in theory be observed in all experimental groups.

Due to the relatively recent emergence of MDPV and related pyrovalerone cathinone derivatives into drug markets, we had no *a priori* hypotheses or predictions prior to the commencement of this study as to possible changes in specific cytokine levels induced by this drug. However, based on its similar mechanisms of action with cocaine, we anticipated observing a generalized pro-inflammatory profile as reviewed elsewhere (Lucerne et al., 2021; Namba et al., 2021). In addition, some recent studies have indicated that specific cytokines and chemokines mediate MDPV-induced behavioral effects. For example, pharmacological blockade of CXCR4 attenuates the conditioned rewarding and locomotor stimulant effects of MDPV (Oliver et al., 2018), while blockade of CXCR4, CCR5, or IL-17A signaling attenuates changes in anxiety-like behaviors evoked by MDPV (Simmons et al., 2022; Inan et al., 2023). However, in the present study, we found no changes in PFC levels of any of these chemokine ligands or their receptors following MDPV intake, although IL-17A was classified into the cytokine module (Module 5) associated with MDPV intake.

Rather, we found that PFC levels of endothelial cell adhesion protein VCAM-1/CD106 were positively associated with MDPV intake. Consistent with this, it has been demonstrated that cocaine, which acts in a similar manner to MDPV as a presynaptic monoamine transporter inhibitor, also up-regulates VCAM-1/CD106 expression *in vitro* (Gan et al., 1999), where it can lead to increased leukocyte trafficking across the blood brain barrier (Dietrich, 2002). Endothelial VCAM-1/CD106 expression has also been shown to be modulated by both dopamine and norepinephrine (Kapper et al., 2002), the two primary neurotransmitters whose signaling is enhanced by the pharmacological actions of MDPV. While not directly examined in the present study, these findings suggest that the positive association between MDPV intake and PFC expression of VCAM-1/CD106 may lead to compromised blood brain barrier integrity in this region, a phenomenon commonly observed following long-term intake of many psychostimulants (Pimentel et al., 2020). Further studies are warranted to explore this possibility.

Another significant observation in the present study was a negative association between MDPV intake and PFC levels of Flt-3 ligand, which was classified into Module 2. This cytokine exerts a number of hematopoietic functions (Lyman et al., 1993) and is structurally related to SCF, a cytokine identified in Module 5. The functions of Flt-3 ligand in the brain are not well characterized, although it has been reported to participate in recruitment of subpopulations of dendritic cells to the central nervous system (Curtin et al., 2006; Anandasabapathy et al., 2011). A negative association of MDPV intake with Flt-3 ligand levels would therefore, unexpectedly, suggest reduced recruitment of dendritic cells to the brain during abstinence from MDPV. However, given the paucity of information on the functions of Flt-3 ligand in the brain, such conclusions at this point are only speculative, and further studies are warranted to explore this possibility.

Finally, we observed no sex differences in MDPV intake, consistent with a prior study examining intake patterns of the similarly acting cathinone derivative α-PVP (Marusich et al., 2022). However, this study did identify a number of drug induced increases in PFC levels of various cytokines, including IL-1α, IL-1β, IL-6, and TNF-α, which were more prominently elevated in males vs. females. However, unlike the present study, these investigators examined brain cytokine levels within 24 h of cessation of drug intake. Therefore, it is possible that sex-dependent changes in PFC cytokine levels were present during the early stages of abstinence in the current study, and additional studies taking such measures at multiple time points following drug intake are warranted.

## Conclusion

In the current study we demonstrate that after 3 weeks of abstinence, rats with a history of binge-like MDPV intake show differential alterations in expression of a unique cluster or modules of cytokines. Within this module, binge-like self-administration of the cathinone derivative MDPV was positively associated with PFC levels of VCAM-1/CD106, and negatively associated with PFC levels of Flt-3 ligand. The functional significance of these changes are currently unknown, though they suggest potential alterations in the maintenance of blood–brain barrier integrity and immune cell trafficking to the CNS. Additional studies are needed to account for any potential contributions of circulating cytokine levels to observed changes in the brain, as well as at additional time points during or after drug intake. Additional studies are also needed to determine how these cytokines are regulated by the neurochemical mechanisms of action MDPV or other psychostimulants (i.e., increased monoaminergic transmission), as well as their downstream functional effects on PFC-mediated cognition and behaviors.

## Data availability statement

The raw data supporting the conclusions of this article will be made available by the authors, without undue reservation.

## Ethics statement

The animal study was approved by Arizona State University – Tempe Campus. The study was conducted in accordance with the local legislation and institutional requirements.

## Author contributions

EN: Data curation, Investigation, Methodology, Writing – review & editing. JL-J: Data curation, Formal analysis, Investigation, Visualization, Writing – review & editing. LH: Data curation, Investigation, Methodology, Writing – review & editing. AA: Writing – review & editing, Visualization. MO: Visualization, Writing – review & editing, Conceptualization, Data curation, Formal analysis, Funding acquisition, Investigation, Methodology, Project administration, Resources, Supervision, Writing – original draft.
